# DOES CAREGIVING MATTER? THE RELATIONSHIPS BETWEEN CAREGIVING EXPERIENCE AND ADVANCE CARE PLANNING IN OLD AGE

**DOI:** 10.1093/geroni/igz038.496

**Published:** 2019-11-08

**Authors:** Huei-wern Shen

**Affiliations:** Graduate Institute of Social Work, National Taiwan Normal University, Taipei, Taiwan

## Abstract

Advanced care planning (ACP) is encouraged as the completion of ACP increases the likelihood for patients to receive their preferable end-of-life care and for caregivers to be less stressed. Common approaches to increase the engagement of ACP target on intervention or information provision to patients in the very late stage of life. Arguing that caregiving experience may influence how people plan their own end-of-life care, the present study focuses on caregiving roles. Using seven waves of data (2002, 2004, 2006, 2008, 2010, 2012, and 2014) from the Health and Retirement Study (HRS), 863 older people who were 65+ and alive in 2012 but passed away prior to 2014 were included in this study to examine the relationships between an individual’s caregiving experience (2002-2012) and his/her completion of ACP (2014). Findings from logistic regression showed that caregiving experience did not influence older adults’ (65+) ACP completion in 2014. When considering different types of caregiving experience (none, spousal, parental) among older cohort (75+), the odds of completing ACP for those with spousal caregiving experience were 2.47 times more likely than those without any caregiving experience. However, no relationship was detected among those with parental caregiving experience. Other factors relating to ACP completion were poorer health, death being expected, death due to cancer, older age and being racial minorities. Practitioners and policymakers could consider encouraging older adults (65+) who provide spousal care to engage in ACP. Future research taking longitudinal approaches to identify reasons behind the existence of the relationship is urged.

